# One‐stage reconstruction of the massive overlying skin and Achilles tendon defects using a free chimeric anterolateral thigh flap with fascia lata

**DOI:** 10.1002/micr.30931

**Published:** 2022-06-18

**Authors:** Tran Thiet Son, Pham Thi Viet Dung, Ta Thi Hong Thuy, Vu Hong Chien, Le Hong Phuc, Le Anh Huy

**Affiliations:** ^1^ Department of Plastic Reconstructive and Aesthetic Surgery Bach Mai Hospital Hanoi Vietnam; ^2^ Department of Plastic Surgery Saint Paul Hospital Hanoi Vietnam; ^3^ Department of Plastic and Reconstructive Surgery Hanoi Medical University Hospital Hanoi Vietnam; ^4^ Department of Plastic Aesthetic Surgery Hue University of Medicine and Pharmacy Hospital Hue Vietnam

## Abstract

**Background:**

Treatment for large defects in the non‐weight‐bearing Achilles tendon and soft tissues remains a reconstructive challenge. The free composite anterolateral thigh flap (ALT) with fascia lata (FL) has been indicated in the single‐stage reconstruction of the Achilles tendon and soft tissue defect and this technique remain some disadvantages, such as the inability to perform primary flap thinning, requiring secondary flap thinning, and the delayed normalization of the range of motion of the ankle joint. The free chimeric ALT flap with FL was introduced as a novel alternative with many advantages in reconstructing the Achilles tendon and soft tissue defects. This paper reports the reconstruction of the massive Achilles tendon and overlying skin defects using free chimeric ALT flaps with FL.

**Methods:**

From June 2017 to October 2020, we performed on a series of 5 patients receiving free chimeric ALT flaps with FL to reconstruct the Achilles tendon and soft tissue defects. The age of patients ranged from 43 to 62 years old. All five patients had full‐layer defects of the Achilles tendon with infection. The sizes of the skin defects ranged from 6 × 4 cm to 12 × 10 cm. The perforators from the descending branch of the lateral circumflex femoral arteries are located using a handheld Doppler. The perforators help to design the outline of the ALT flap and fascia flap. The skin flap was thinned under microscopy if the flap was too thick. The anastomosis was accomplished before insetting the flaps into the defect.

**Results:**

The size of the ALT flap ranged from 10 × 5 cm to 15 × 12 cm, and the size of the FL flap ranged from 7 × 4 cm to 10 × 8 cm. The mean perforator length for the skin flap and fascia lata was 3.3 cm (range, 2.5–5.0 cm) and 5.3 cm (range, 3.5–7.0 cm), respectively. Four patients received skin flap thinning up to 57%–79% of the flap thickness, while one patient did not need to debulk. The thickness of the ALT flap ranged from 6 to 13 mm. All the flaps survived completely and postoperative courses were uneventful without any complications. The follow‐up time ranged from 12 to 51 months. All patients were able to stand and ambulate, and they were satisfied with the reconstructive results.

**Conclusions:**

The free thin chimeric ALT with FL flap is appeared to be an appropriate treatment for the massive Achilles tendon and overlying skin defects. This may be a practical approach to improve the functional outcomes of patients with infected massive Achilles tendon and overlying skin defects.

## 1 INTRODUCTION

Achilles tendon and overlying skin defects are always a reconstructive challenge, especially large defects caused by major trauma, cancer, or burns (Kellam et al., 1985; Strauss et al., 2007). In addition, the improper initial treatment causes infection or necrosis of the Achilles tendon area, leading to more complex reconstructive processes. Several reconstructive approaches were applied to treat Achilles tendon rupture and overlying skin defects, but their results were limited (Ahmad et al., 2016; Hanada et al., 2011). Free flaps were suitable for simultaneous reconstruction of the Achilles tendon and complex soft tissue defects (Fischer et al., 2013). In 1984, the anterolateral thigh (ALT) flap described by Song et al. became the preferred alternative for skin defect reconstruction (Song et al., 1984), especially in lower extremity soft tissues (Lee et al., 2000; Lee & Lee, 2018; Nosrati et al., 2012). In 1990, the use of ALT flaps with vascularized fascia lata in Achilles tendon rupture was first reported by Inoue et al. (Inoue et al., 1990). The free composite ALT flap with FL has advantages in the single‐stage reconstruction of the Achilles tendon and soft tissue defect. However, available techniques have many disadvantages, leading to suboptimal clinical and esthetic patient outcomes and a longer recovery time/healing process. A major disadvantage is the inability to perform primary flap thinning, requiring secondary flap thinning, and the delayed normalization of the range of motion of the ankle joint (Andreu‐Sola et al., 2017; Duhamel et al., 2010; Houtmeyers et al., 2012; Kuo et al., 2003; Michel et al., 2014; Youn et al., 2015).

The free thin chimeric ALT flap with FL was introduced as a novel alternative technique that is hypothesized to overcome such disadvantages and provide better patient outcomes with a shorter recovery time, especially for patients with Achilles tendons and complicated skin defects by infection. However, there has been no report on using a free thin chimeric ALT flap with FL to reconstruct the massive Achilles tendon defects and cover damaging skin. This report aims to share our experiences about the long‐term outcomes of patients with massive infected Achilles tendon and overlying skin defects who underwent the free thin chimeric ALT flap in single‐stage reconstruction.

## 2 PATIENTS AND METHODS

From June 2017 to October 2020, five patients underwent reconstruction of the Achilles tendon and skin defects using the free ALT flaps. There were 2 male patients and 3 female patients between the ages of 43 and 62. One patient had an ulcerated basal cell carcinoma and one patient was bitten by a monkey; the rest were all in motorcycle accidents. All patients had Achilles tendon area injuries and were subsequently treated in other hospitals and underwent multiple surgical procedures, such as primary repairs, multiple debridements, local flap reconstruction, vacuum‐assisted closure (VAC), and skin graft. All the patients ultimately presented to our hospital with severe wound infection, resulting in full‐layer defects of the Achilles tendon. Staphylococcus aureus was identified in bacterial culture in one patient, while Pseudomonas aeruginosa was identified for the rest of the patients. The period from initial injury to treatment at our hospital ranged from 4 to 12 weeks. The mean prior surgical procedures were 3.2 times (range, 2–5 times). During hospitalization up to 4 days postoperatively, the patients received cefazolin 2 g IV a day for Staphylococcus aureus and gentamicin 1.5 mg/kg IV plus vancomycin 1 g IV a day for Pseudomonas aeruginosa. VAC wound management was used for 7 days prior to surgery. The sizes of the overlying skin defects ranged from 6 × 4 cm to 12 × 10 cm, and the length of Achilles tendon defects ranged from 4 to 6 cm.

### 2.1 Surgical technique

We planned to use a chimeric ALT flap and FL flap to reconstruct Achilles tendons and soft tissue defects simultaneously. The patients were placed in a supine position, a straight line was drawn between the anterior superior iliac spine and the superolateral border of the patella on the donor's thigh. The line was divided into 16 parts, and a circle with a 2.5 cm radius was drawn around the midpoint. Next, we used a handheld Doppler to locate and map the perforators to mark the skin. We designed an appropriate outline of the skin paddle for the skin defect.

One surgical team started the operation with an incision from the medial border of the skin flap to the subfascial plane. The dissection continued in the subfascial plane until the descending branch of the lateral circumflex femoral artery (LCFA) and its perforators were identified. We then chose two perforators for the chimeric flap, including the skin flap and the FL flap, both perforators coming from the descending branch. Based on the size of the skin defect, we harvested ALT and FL flaps with the independent perforators. The skin flap was thinned under microscopy if the flap was too thick, the fat under the superficial fascia was removed by blunt scissors, and subdermal vessels were preserved. To effectively reconstruct the Achilles tendon, the FL flaps were taken approximately 2 cm longer than the length of the defect of the Achilles tendon. The ALT flap and FL flap were elevated from the thigh and their pedicle was cut from the descending branch of LCFA.

A second surgical team debrided the necrotic tissues. Dissections were made to the posterior tibial artery and venae comitantes, then an end‐to‐end or side‐to‐end anastomosis was performed to connect the flap pedicle and the posterior tibial vessel. The anastomosis was accomplished before insetting the flaps into the defect. After confirmation of blood perfusion, the Achilles tendon and skin defect reconstructions were performed. The FL flaps were folded 2 or 3 times to create a tendon‐like structure, and then it was subsequently attached to the healthy remnants of the Achilles tendon using 2–0 prolene simple interrupted sutures or to the calcaneus bone using screws. Finally, the skin defect was covered with the thinned skin paddle flap over a silicone drain. All the donor sites were primarily closed. Postoperatively, the ankle was immobilized in the neutral position with an above‐knee splint for 3 weeks.

## 3 RESULTS

The detailed information and reconstruction results are shown in Table 1. The size of the ALT flaps was from 10 × 5 cm to 15 × 12 cm, while the size of the FL flaps ranged from 7 × 4 cm to 10 × 8 cm. The mean length of the descending branch was 8 cm (range, 7–9 cm). The mean length of the perforator for the skin flap was 3.3 cm (range, 2.5–5.0 cm), while the mean length of the perforator for FL flap was 5.3 cm (range, 3.5–7.0 cm). Four patients received skin flap thinned from 57 to 79% of the flap thickness, and one did not need this procedure. The mean thickness of the skin flap was 8.8 mm (range, 6–13 mm). The Achilles tendon defects were reconstructed with triple‐folded fascia sheets in 3 patients, and the 2 others with double‐layer fascia sheets. In four patients, we attached the FL to the healthy proximal remnant of the Achilles tendon on both sides by sutures. For the last patient, FL was attached to the healthy proximal remnant on one side, and the calcaneus bone by screws on the other side. The descending branch was anastomosed with the posterior tibialis vessels in a side‐to‐end fashion in 3 patients and an end‐to‐end fashion in 2 patients. All the flaps survived completely without complications. The flap donor site was primarily closed without related morbidities, and wound healing was uneventful. All patients received physical therapy of the ankle joint starting at the 3rd week postoperatively. The mean follow‐up was 29.4 months (range, 12–51 months). The mean arc of ankle rotation between dorsiflexion and plantar flexion was 54° (range, 50°‐60°). All patients were able to wear shoes, stand, and ambulate without further debulking procedures. They were satisfied with the esthetic outcomes.

**TABLE 1:** General characteristics and outcomes of patients

Pt.	Sex/ age (year)	BMI	Etiology	Time injury (week)	Infection	Previous surgical procedures time of procedure	Size of STD (cm)	Size of ALT flap (cm)	P1 (cm)	Thickness of flap before / after thinning (cm)	Size of FL flap (cm)	P2 (cm)	Type of layer fascia sheets	DB (cm)	Type of anastomosis	Follow‐up (month)	AAR
1	M/52	20.8	Basal cell carcinoma ulcer	12	Sta. Aureus	Debridement and VAC 5	8 × 5	19 × 7	3.0	1.3	10 × 8	4.5	Triple	7	PTA Side to end	51	55^0^
2	M/43	25.1	Monkey bite	5	Pseu. Aeruginosa	Primary repair incision debridement and VAC 3	10 × 7	12 × 8	3.0	1.5/0.6	10 × 6	4.5	Triple	9	PTA Side to end	48	60^0^
3	F/59	24.7	Motorcycle accident	4	Pseu. Aeruginosa	Primary Achilles tendon repair 2	12 × 10	15 × 12	3.0	2.8/0.6	12 × 6	7.0	Triple	9	PTA End to end	24	50^0^
4	F/54	26.3	Motorcycle accident	4	Pseu. Aeruginosa	Primary Achilles tendon repair and VAC 2	6 × 8	10 × 9	5.0	3.2/0.7	7 × 4	7.0	Double	8	PTA End to end	12	55^0^
5	F/62	24.0	Motorcycle accident	7	Pseu. Aeruginosa	Primary Achilles tendon repair and debridement 4	6 × 4	16 × 7	2.5	2.8/1.2	8 × 5	3.5	Double	7	PTA Side to end	12	50^0^

Abbreviations: AAR, Arc of ankle rotation between dorsiflexion and plantarflexion; ALT, Anterolateral thigh flap; BMI, Body Mass Index; DB, Length of descending branch; F, Female; FL, Fascia Lata; M, Male; P1, Perforator to ALT flap; P2, Perforator to Fascia Lata flap; Pt., Patient number; PTA, Posterior tibial artery; STD, Soft Tissue Defect; TAD, Tendon Achilles Defect; VAC, Vaccum Assisted Closure.

## 4 CASE REPORTS

### 4.1 Case 1

**FIGURE 1:**
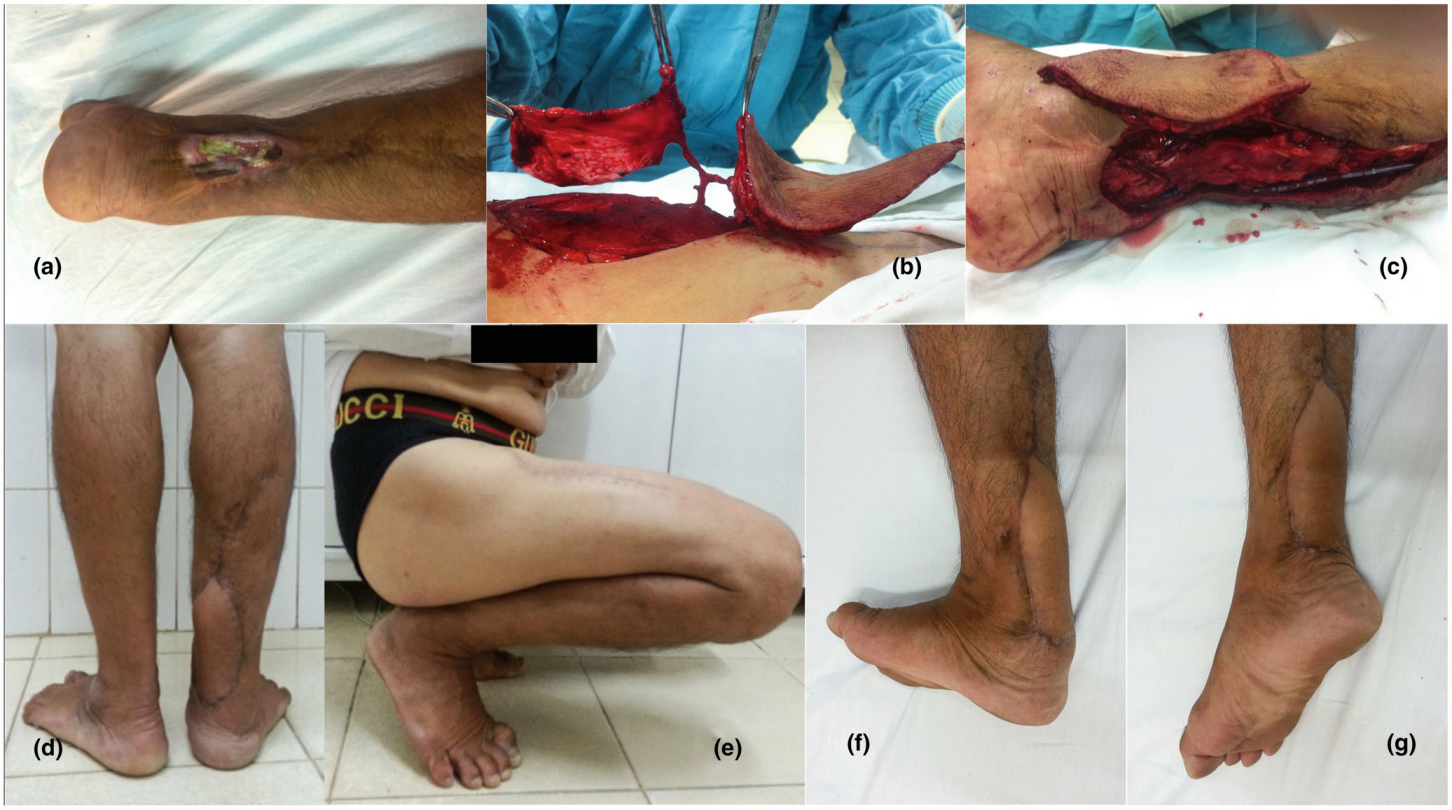
A 52‐year‐old man presented with basal cell carcinoma of the right ankle on a 40‐year‐old chronic scar. (a) Appearance of the necrotic Achilles tendon and soft tissue defect before debridement and VAC. (b) A chimeric ALT flap skin and fascia lata flap were harvested. (c) The wrapping of newly reconstructed Achilles tendon with fascia lata. (d) After 18 months of follow‐up, he was able to stand. (e) The patient was able to squat. (f) Dorsiflexion was 15°. (g) Plantar flexion is 40°.

A 52‐year‐old male patient presented with necrosis of the Achilles tendon and a skin defect due to a war injury in the right lower leg 40 years ago. About 4 years before visiting our hospital, he began to feel pain, occasionally bleeding from an ulcer, and within last year, the ulcer had expanded and deepened with bleeding and purulent discharge. When he was admitted to our hospital, a 4 × 5 cm ulcer with green purulent exudate and red swelling at the margin was found in the posterior 1/3 of the lower leg. Staphylococcus aureus was identified in bacterial culture and the patients received cefazolin 2 g IV a day. The size of the skin and the Achilles tendon defects were 8 × 5 cm and 5 cm, respectively (Figure 1a). A biopsy of the ulcerative lesion was obtained and the result came back positive for basal cell carcinoma. An ALT flap with FL was prepared, the location of the two perforators from the descending branch of LCFA was identified with Doppler ultrasonography and the skin was marked. After dissecting the 2 perforators and the descending branch pedicle, the skin paddle flap measuring 19 × 7 cm with a thickness of 13 mm and the FL flap measuring 10 × 8 cm were harvested (Figure 1b). Anastomosis of the posterior tibial arteries to the descending branch was performed in a side‐to‐end fashion. The Achilles tendon was reconstructed with the triple‐folding FL flap with the Pulvertaft technique (Figure 1c). The skin defect was reconstructed with the ALT flap. The patient was discharged 14 days postoperatively without complications. The patient was able to ambulate 2 months after the operation, and after 18 months of follow‐up, the patient's functions and esthetic results were achieved in both donor and recipient sites (Figure 1d). He was able to squat and walk normally (Figure 1e), and performed an ankle rotation of 15° with dorsiflexion and 40° plantar flexion (Figure 1f,g).

### 4.2 Case 2

**FIGURE 2:**
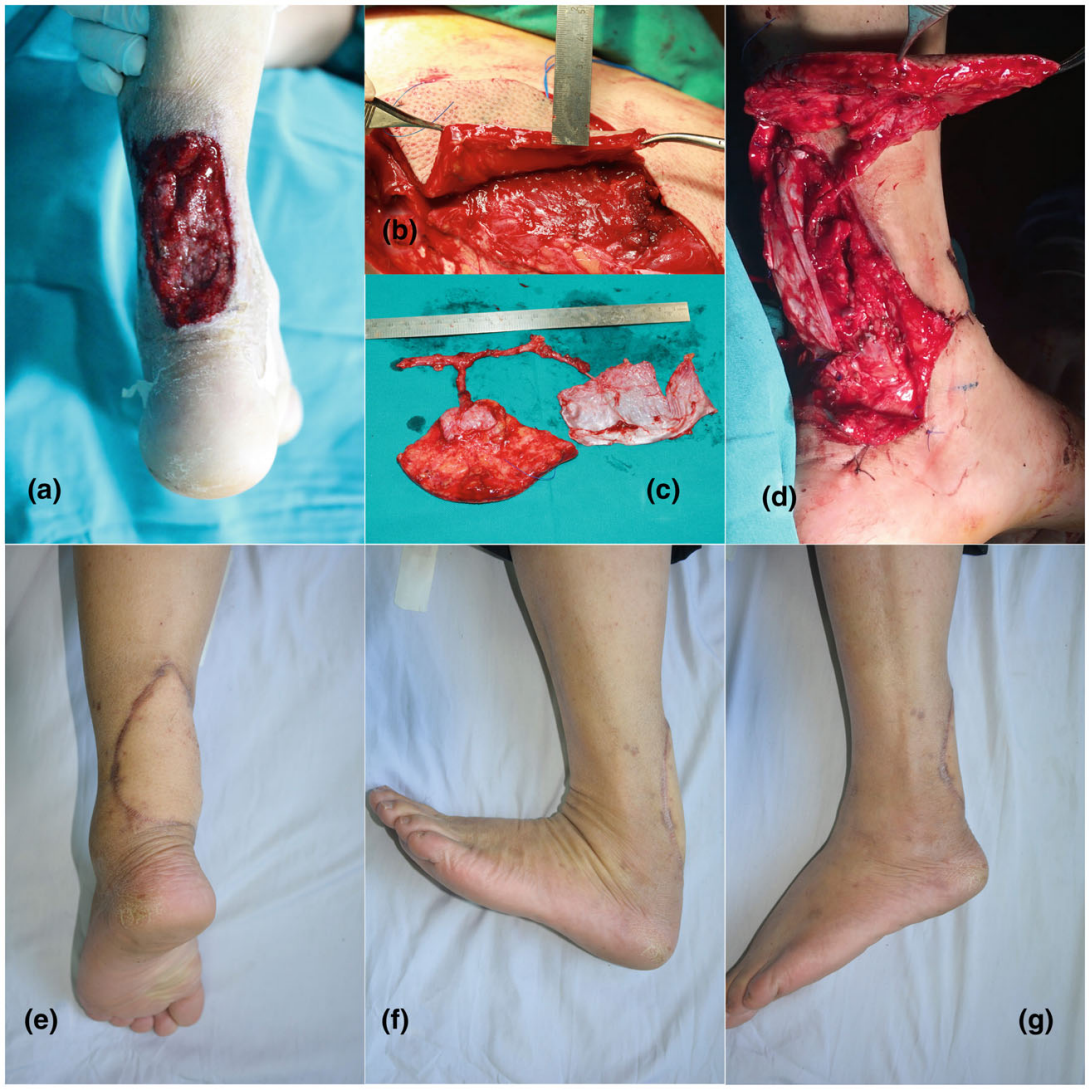
A 43‐year‐old man with a left ankle injury by a monkey 5 weeks ago and underwent 2 primary repair procedures at a local clinic. (a) The overlying soft tissue defects measured 5 × 10 cm after debridement. (b) A skin flap was thinning down to 6 mm was harvested. (c) The chimeric ALT skin flap with FL flap. (d) The fascia was folded to form a triple‐layer tendon. (e) After 2 years of follow‐up. (f) Dorsiflexion was 15 degrees. Plantar flexion was 45 degree.

A 43‐year‐old male patient presented with a left ankle injury from a monkey bite and he was admitted to a provincial hospital for treatment with sutures and antibiotics. However, his wound became infected. The patient was referred to our hospital with a wound infection caused by Pseudomonas aeruginosa. Antibiotics gentamicin 1.5 mg/kg IV plus vancomycin 1 g IV once daily were given. After 7 days of VAC wound management, he underwent surgical debridement, which revealed large skin and Achilles tendon defects measuring 7 × 10 cm and 3 × 6 cm, respectively (Figure 2a). An 8 × 12 cm ALT flap was dissected. Skin paddle thickness was 15 mm; then, it was thinned down to 6 mm (Figure 2b). Two perforators were identified, including a perforator of 3 cm for the ALT flap and another perforator of 4.5 cm for the FL flap. Both perforators originated from the descending branch of LCFA (Figure 2c). We performed an end‐to‐side anastomosis between the posterior tibial and descending branches. The FL flap with the size of 6 × 10 cm was triple‐folded and sutured to the Achilles tendon at both ends (Figure 2d). The ALT flap was covered with skin defects and survived completely without complications. The patient was able to resume ambulation 4 weeks postoperatively. After 24 months of follow‐up, we observed that patient showed an uneventful arc of ankle rotation (Figure 2e). The ankle rotation was 15° with dorsiflexion and 45° plantar flexion (Figure 2f,g). The patient was able to walk and jog normally.

### 4.3 Case 3

**FIGURE 3:**
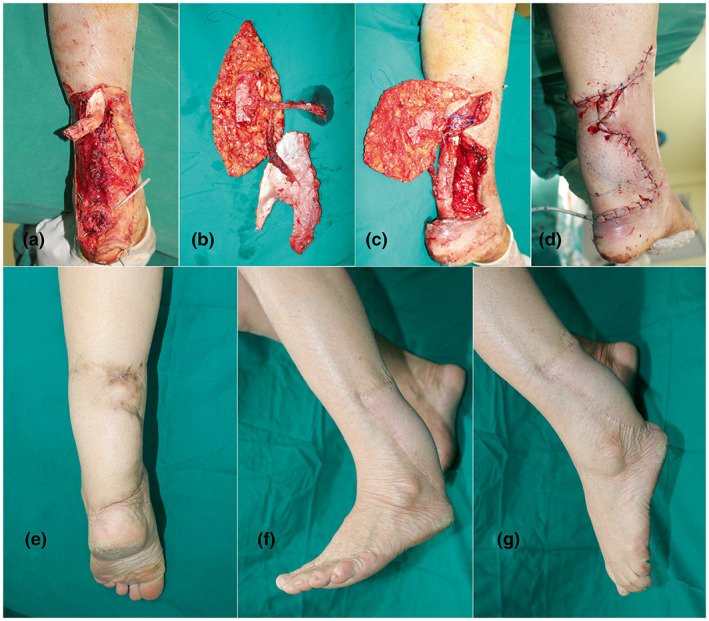
A 59 year‐old woman presented with an Achille tendon injury and extensive soft tissue necrosis on the ankle as a result of a motorcycle accident 4 weeks ago. (a) Appearance of necrosis of Achilles' tendon and soft tissue defect after radical debridement. (b) View of the chimeric ALT flap after thinning. (c) The fascia was folded to form a triple‐layer neo‐Achilles tendon. (d) Immediate postoperative view. (e) View the operative ankle after 18 months of follow‐up. (f) Dorsiflexion was 10 degrees. (g) Plantaflexion was 40 degrees.

A 59‐year‐old woman presented with an Achilles tendon rupture and a large skin defect on the right leg due to a motorbike accident. She was treated at another health facility with Achilles tendon reattachment and skin suturing, but the wound produced discharge and necrosis of soft tissues developed. The patient came to us with necrosis of the Achilles tendon and the skin of the heel (Figure 3a). The wound culture was positive for Pseudomonas aeruginosa, so gentamicin 1.5 mg/kg IV plus vancomycin 1 g IV once daily and VAC management were given to treat the infection. A 12 × 15 cm skin flap was planned and dissected, and two perforators were identified, including a 3 cm long perforator for the skin flap and another 7 cm long perforator for the FL flap. The two perforators originated from the descending branch of LCFA. The thickness of the skin paddle has been thinned from 28 mm to 6 mm. The harvested descending branch measured 9 cm (Figure 3b). The 12 × 6 cm FL flap was a triple‐folded of structure the tendon, and it was sutured to the Achilles tendon at one end, and the calcaneus at the other end (Figure 3c). The thinned ALT flap was then used for skin defect coverage (Figure 3d). The patient was able to resume ambulation 6 weeks postoperatively. The follow‐up after 24 months showed an uneventful arc of ankle rotation, with dorsiflexion 10° and plantar flexion 40° (Figure 3e–g). The patient could walk and jog normally.

## 5 DISCUSSION

The Achilles tendon plays an important role in maintaining the balance and transmitting forces of the body during movement. Therefore, ruptures of the Achilles tendon will severely affect the patient's walking function. Due to the heavy load on the Achilles tendon, the surrounding area with relatively poor vascularity and high susceptibility to infections, reconstruction of the Achilles tendon is always a surgical challenge. Furthermore, inappropriate treatment of the Achilles tendon can cause infection and thus make the wound condition more complex, resulting in extensive tendon defects and overlying skin necrosis (Fourniols et al., 2012; Molloy & Wood, 2009). Infections are usually caused by Pseudomonas aeruginosa, Staphylococcus aureus, and Streptococcus (Molloy & Wood, 2009; Strauss et al., 2007), and we also faced the problem of infection in this report. Intravenous antibiotic therapy is indicated preoperatively, intraoperatively, and postoperatively (Strauss et al., 2007). Infected necrotic skin and tendon tissue were resected and a wound bed was prepared preoperatively with negative‐pressure therapy for reconstruction with flaps (Fourniols et al., 2012; Repta et al., 2005). The ideal reconstructive option is free tissue transfer and various types of composite free flaps have been proposed for small defects in the Achilles tendon region, including muscle flap, musculocutaneous flap, fasciocutaneous flap, and perforator flap (Abhyankar et al., 2009; Berthe et al., 1998; Huemer et al., 2012; Kim et al., 2003; Lee et al., 1999; Papp et al., 2003; Smit et al., 2012; Yajima et al., 2001; Yuen & Nicholas, 2001). The ALT flap with vascularization source from perforators of the descending branch of LCFA paved the way for the use of new materials to reconstruct large skin defects or chronic wound infections at the ankle (Lee et al., 2000; Lee & Lee, 2018; Nosrati et al., 2012). Several authors presented the application of composite free ALT flap with the FL to reconstruct the complicated Achilles tendon and skin defects. In this technique, the vascularized FL flap offers optional benefits such as early and rapid healing, high infection resistance, increased tendon mobility, and single‐stage surgery (Andreu‐Sola et al., 2017; Duhamel et al., 2010; Houtmeyers et al., 2012; Inoue et al., 1990; Kuo et al., 2003; Michel et al., 2014; Youn et al., 2015). However, the disadvantages of the free composite ALT flap are to have pedicle instability and the flap being too thick.

The chimeric flap is considered an alternative to improve the shortcomings of a combined free ALT flap. Chimeric flaps present advantages in complex reconstructive planning due to their large skin area and reliability, increased freedom of inset, reduced operative time, minimized morbidity, and improved functional outcomes (Kim, Kim, & Ghanem, 2015; Koshima et al., 1993). Chimeric free flaps were used to reconstruct the lower extremity (Kim, Kim, Hong, et al., 2015; Song et al., 2016; Zheng et al., 2016). To date, no study has reported the use of free chimeric ALT flap for complex Achilles tendon and skin defect reconstruction. In all cases of our report, at least 2 perforators derived from descending branch of LCFA were found to supply the skin and FL flap. We prefer to select the larger and more distal perforating vessel for the skin flap. The FL sheet design is performed along the thigh axis. As the FL did not adhere to the skin paddle, the tendon could be easily reconstructed. Chimeric free ALT flaps have several advantages over using composite free ALT with FL, such as a well‐vascularized fascial flap supplied by a perforator, tendon reattachment after the vascular anastomosis is easy to perform as the FL flap can be folded into a tendon‐like structure. In addition, the skin flap allows extensive surgical maneuvers, and a free‐style skin flap increases the gliding surface for tendon movement. Another significant advantage of the chimeric flaps is that flap debulking can be easily performed as the skin flap separates from the FL flap without the risk of damaging the vascular supply to both skin and FL flaps.

For reconstruction of the Achilles region, the ideal flap should be thin and pliable for molding the ankle ‐ heel contour. The composite ALT flaps are too bulky, making them esthetically and functionally unfavorable for ankle reconstruction. To achieve better esthetic results, most composite free ALT flaps require secondary thinning after reconstruction to remove fat using scissors or aspiration. Secondary thinning increases the tendency to create adhesion of the overlying skin (Agostini et al., 2013). The primary thinning or the microdissected thinning was performed before flap transfer and these procedures can reduce the thickness of skin flaps down to approximately 3–12 mm (Kimura et al., 2001; Kimura et al., 2003). The flap thinning has the advantage of creating less bulky tissue, eliminating the need for subsequent thinning procedures, and providing higher quality skin for coverage, which promotes quicker ankle mobility. In our series, the ALT flap was thinned in 4 patients by microdissection, and the flap thickness was reduced by 57%–79%. This is equivalent to 6–12 mm, and all thinned flaps survived well. A patient, who had an extracted flap thickness of 13 mm, did not need to undergo thinning. Controlled ALT flap thinning can only be successfully performed using a chimeric fashion, and cannot be applied to composite ALT flap. Furthermore, we consider skin flap thinning is required to produce better esthetic outcomes, especially for female patients. Follow‐up after surgery showed that all reported patients in our report who underwent free ALT with FL transfer to the non‐weight‐bearing Achilles tendon region had significantly shorter recovery periods than those in other studies. Additionally, the rates of complications or revision surgeries (flap contouring or debulking) have not occurred as in other studies (Grauberger et al., 2020). All patients received adequate length of hospital stay and treatment, reducing the time needed to begin rehabilitation. The patients were able to resume ambulation 4–8 weeks postoperatively, complete their normal daily tasks, and walk at 2 months postoperatively. All patients were “satisfied” with the esthetic appearance of their reconstruction.

The limitations of this report depend on the number of perforators obtained during dissection. The disadvantage of the ALT flap is the unfavorable variations of perforators supply to the ALT flap. The incidence of perforator absence is about 0.89% to 5.4%. Only 45%–100% of the perforating branches originate from the descending branch (Kawai et al., 2004; Lakhiani et al., 2012). Therefore, in a significant number of patients, it is not possible for more than 2 perforators to arise from the descending branch, and thus, no chimeric flaps can be created. In cases where only one perforator can be dissected, the best option is to use a composite ALT flap with the fascial flap attached to the skin flap. If a perforator from the descending branch of LCFA is present, reconstruction with a free fabricated chimeric flap is the best option in which the single perforator supplies blood to the skin flap, and a FL flap can be harvested from the contralateral thigh. Therefore, the descending branch is first anastomosed with the posterior tibialis vessels, then the pedicle of the FL flap is anastomosed with a side branch of the descending branch of LCFA, as introduced by Ando in 2019 (Ando et al., 2019). The disadvantage is that the surgical procedure is rather complicated and difficult due to multiple vascular anastomoses.

## 6 CONCLUSION

The free chimeric ALT flap with FL appeared to be an appropriate approach to reconstructing the complicated soft tissue and Achilles tendon defects among patients included in this study. In addition, the thinning of the skin flap was shown to improve the functional and esthetic outcomes of surgical reconstruction. This study provides preliminary evidence indicating that the thinning chimeric ALT free flap might offer a safe, reliable, and esthetically appealing option for a single‐stage Achilles tendon reconstruction, including the overlying soft tissue defects. A larger study is needed to understand better and quantify the benefits, patient outcomes, and challenges of this novel surgical technique for patients who suffer complex soft tissue and Achilles tendon defects,

## CONFLICT OF INTEREST

The authors declare no conflict of interest.

## Data Availability

Data sharing not applicable to this article as no datasets were generated or analysed during the current study.
